# Repair of Mutated *NF1* mRNA with Trans-Splicing Group I Intron Ribozymes

**DOI:** 10.3390/cancers17172749

**Published:** 2025-08-23

**Authors:** André Leier, Xu Han, Jehanne Aghzadi, Erik Westin, Jian Liu, Tatiana T. Marquez Lago, Robert A. Kesterson, Bruce R. Korf, Deeann Wallis, Ulrich F. Müller

**Affiliations:** 1 Department of Genetics, University of Alabama at Birmingham, Birmingham, AL 35294, USA; erik.westin@pbrc.edu (E.W.); jianliu@uabmc.edu (J.L.); tmarquezlago@uabmc.edu (T.T.M.L.); robert.kesterson@pbrc.edu (R.A.K.); bkorf@uabmc.edu (B.R.K.); dwallis@uab.edu (D.W.); 2 Department of Chemistry & Biochemistry, University of California San Diego, La Jolla, CA 92093, USA; xuhan@ucsd.edu (X.H.); jehanne.aghzadi@univ-amu.fr (J.A.)

**Keywords:** group I intron, ribozyme, *Tetrahymena thermophila*, trans-splicing, extended guide sequence, Neurofibromatosis Type I, *mNf1*, *hNF1*

## Abstract

Background/Objectives: Therapeutic strategies for Neurofibromatosis Type I (NF1) that correct the underlying pathogenic *NF1* variant hold promise for restoring neurofibromin function, reducing tumor burden, and improving patient outcomes by addressing the root cause of the disease rather than its symptoms. Beyond gene editing, transcript reprogramming via RNA trans-splicing has gained attention, particularly with the recent FDA approval of two trans-splicing-based drugs for IND phase 1/2a trials. This study tests whether trans-splicing group I intron ribozymes from *Tetrahymena thermophila* can be used to repair pathogenic variants of *NF1* (pre-)mRNA by 3′-tail replacement. Methods: Splice sites on the *NF1* mRNA were identified computationally and validated biochemically, and an efficiency-enhancing Extended Guide Sequence (EGS) of the corresponding ribozyme was identified in a combinatorial experiment. Results: The correct trans-splicing product of this ribozyme was validated in HEK293 *NF1−/−* cells expressing *mNf1*. Conclusions: This study established a splice site and activity-enhancing extended guide sequences for the repair of NF1 mRNA. Further optimization of the ribozyme, as well as improved delivery methods, may establish ribozyme-based RNA repair as a viable strategy for NF1 treatment.

## Simple Summary

This study shows that a portion of mutated *NF1* (pre-)mRNA can be corrected in human cells using trans-splicing ribozymes. To do so, the position *mNf1* c.6580 (NM_010897.2; r.6688), corresponding to *hNF1* c.6574 (NM_001042492.3, r.6907), was identified computationally as a promising splice site for 3′-end replacement and validated by in vitro splicing reactions. Trans-splicing efficiency was further increased in a combinatorial experiment that determined an improved extended guide sequence (EGS) for the ribozyme. When this ribozyme was transfected into HEK293 *NF1−/−* cells expressing full-length *mNf1* cDNA, the correct *mNf1* trans-splicing products were identified.

## 1. Introduction

Neurofibromatosis Type 1 (NF1, OMIM #162200) is an autosomal dominant genetic disorder caused by pathogenic variants in the *NF1* gene (NCBI Gene ID 4763), which encodes the tumor suppressor protein neurofibromin. Over 3000 distinct pathogenic variants of this gene have been identified, which are spread across the entire length of the gene with no obvious hotspot [1]. NF1 affects approximately 1 in 3000 individuals worldwide, though recent studies leveraging a genotype-first approach demonstrate an unexpectedly high prevalence of 1 in 1286 [2]. NF1 is characterized by features such as neurofibromas, café-au-lait spots, Lisch nodules, learning difficulties, skeletal abnormalities, and an increased risk of certain tumors [3,4,5,6]. However, the NF1 phenotype is diverse and variable, even among patients with identical variants [7]. Current treatments for NF1 primarily focus on managing symptoms rather than addressing the underlying genetic cause. Therapeutic strategies that restore *NF1* gene function—whether at the DNA, RNA, or protein level—are not yet available but could be transformative for NF1 treatment [8]. Recent data indicates that restoration of *NF1* expression can normalize mouse sciatic nerves [9]. Here, we describe the repair of mutant *NF1* transcripts using a ribozyme (catalytic RNA).

**Figure 1:**
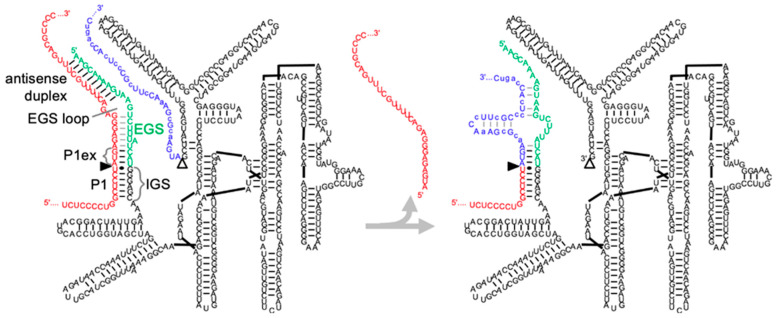
Secondary structure schematic of a trans-splicing *Tetrahymena* group I intron ribozyme before and after a trans-splicing reaction. The *mNf1* target RNA (red) is recognized by the ribozyme’s internal guide sequence (IGS) forming the P1 helix (P1). The extended guide sequence (EGS; green) extends the secondary structure formed with the target RNA to form a 3-base pair extension of the P1 helix (P1ex), an antisense duplex, and in this particular case an EGS loop that is partially closed (gray base pairs). After the target splice site (filled triangle) is cleaved, the ribozyme catalyzes a nucleophilic attack of the substrate’s new 3′-terminus at this splice site to the phosphate linking the ribozyme (black) to its 3′-exon (blue) at the 3′-splice site (empty triangle).  Together, these two catalytic steps replace the substrate 3′-exon with the ribozyme’s 3′-exon. The reaction product contains the substrate 5′-portion (red) and the ribozyme’s 3′-exon (blue) to form the repaired RNA (see structure on the right). The specific sequences show the best-performing sequences of this study, with the chosen *mNf1* splice site 6580, the EGS 22, and the first 30 nucleotides of the ribozyme’s 3′-exon with silent mutations indicated as lower-case letters. The secondary structure [35] is shown with the P4–P6 domain moved to the right for clarity.

Group I intron ribozymes are pre-mRNA introns that do not require the spliceosome for their removal [10]. Instead, these introns fold into three-dimensional structures and catalyze two transesterification reactions that result in their excision, and the joining of the flanking exons. Group I intron ribozymes were first identified in the species *Tetrahymena thermophila* [10], and the ribozyme from this species is among the most studied and manipulated ribozymes [11,12,13,14,15]. The *Tetrahymena* group I intron ribozyme has been re-designed to act in trans, by converting the cis-acting splice site recognition duplex at the ribozyme’s 5′-terminus to a trans-acting duplex [16] (Figure 1). In this format, the ribozyme can recognize a splice site on a substrate RNA and replace the 3′-portion of the substrate with the ribozyme’s own 3′-exon. If the 3′-portion of the substrate RNA carries a disease-causing mutation then the ribozyme is able to replace the mutated sequence with the correct sequence and thereby restore the expression of functional protein [16].

Trans-splicing group I intron ribozymes have been explored for mRNA repair across various genetic diseases. For instance, mutations in the mRNAs of beta globin and *DMPK* were repaired to address the heritable genetic diseases sickle cell anemia [17] and myotonic muscular dystrophy [18]. Aberrant mRNAs of p16 [19] and p53 [20,21] were repaired as an anti-cancer strategy. More recently, a *Tetrahymena thermophila*-derived trans-splicing ribozyme became the first of its kind to receive FDA approval for Phase I/IIa IND trials for hepatocellular carcinoma and glioblastoma [22,23,24]. While this drug does not repair mRNA—it reprograms substrate RNA to induce tumor cell death [25,26,27,28]—it is a milestone for the use of trans-splicing group I intron ribozymes in therapeutic applications.

Two sequences on the *Tetrahymena* group I intron ribozyme need to be adjusted to establish a trans-splicing ribozyme for a specific target mRNA (Figure 1). First, the internal guide sequence (IGS) is a six-nucleotide sequence near the ribozyme’s 5′-terminus. This IGS specifies the splice site on the target RNA by forming the P1 duplex with the target site. The P1 duplex includes a G:U pair between a ribozyme’s G and the splice site U, and five adjacent base pairs with the substrate mRNA. In principle, any uridylate in the target RNA can serve as splice site—but most Us are not easily accessible due to secondary structure formation in the substrate. Accessible Us can be identified by a computational procedure that computes the free energies of three different secondary structures [29], followed by experimental validation of the identified splice sites. Second, extensions of the ribozyme’s 5′-terminus past the splice site can strongly increase splicing efficiency [30]. This extension is called the Extended Guide Sequence (EGS) and consists of 3 nucleotides that extend the P1 helix in the ‘P1 extension’ (P1ex), an internal loop, and an ‘antisense duplex’ between the ribozyme’s 5′-terminus and the substrate. The length requirement of the antisense duplex differs between studies, where antisense duplexes of 35 and 46 base pairs were chosen in the final constructs [30,31], while we found that these duplexes do not need to be longer than 8 base pairs if the splice sites were well accessible [32,33,34]. It is currently unclear what sequences in the internal loop region mediate most efficient splicing. While our previous combinatorial approach found that the EGS side of the loop should be 3–6 nucleotides longer than the substrate side [32], it remained unclear what should be the sequence and possible structure formation of this loop, as well as possible formation of a P10 helix between this EGS loop and the ribozyme’s 3′-tail [34]. These open questions on the EGS loop are important because a single mutation even in a well-designed EGS can further enhance trans-splicing efficiency by over 50-fold [33].

Here we describe the development of a trans-splicing group I intron ribozyme that targets *mNf1* mRNA at position 6580. This splice site was determined by a computational prediction followed by experimental testing in vitro. The ribozyme’s EGS was chosen after reacting a ribozyme library with randomized EGSs with a truncated *NF1* target RNA and identifying suitable EGSs via a barcode in the ribozyme’s 3′-tail. Biochemical analysis of the most promising EGSs showed highest activity for EGS 22. The ribozyme with EGS 22 was tested in HEK293 cells, and trans-splicing was shown by the isolation of RNA and determining the splice product sequence with the correct splice junction. The results are discussed in the context of ribozyme-based *NF1* RNA repair, and possible next steps towards a therapeutic NF1 application are discussed.

## 2. Materials & Methods

### 2.1. Computational Prediction of Free Binding Energies at Different Splice Sites

The computational prediction of promising splice sites on *NF1* mRNA was based on a published method [29] with several modifications. This method calculates the sum of three free folding energies, namely the energy that is required to unfold the target (*NF1* mRNA), the energy required for the ribozyme’s internal guide sequence to be unfolded, and the energy released by hybridization between ribozyme and substrate (formation of the P1 helix). The resulting free energy is the free energy of substrate binding by the ribozyme, and a good predictor of trans-splicing efficiency [29]. Since each U in the target RNA is a possible splice site, the substrate binding free energy was computed for all uridylates in the region of interest on *NF1* mRNA. Here, the binding energies were computed for all uridylates downstream of position 4500 in human *NF1*‘s coding region (NM_001042492.3; transcript variant 1). For each splice site U in the substrate sequence at position *p*, the corresponding ribozyme sequence consisted of a 6-nucleotide long IGS, a 3-nucleotide long P1ex, the ribozyme body, and the corresponding 3′ exon replacement sequence. The IGS and the P1ex are reverse complementary to the substrate sequence from position *p*−5 to *p+*3. The P1ex was not used in [29] but appeared appropriate here because the ribozymes were also tested with such P1 extensions. Binding energies were calculated using the software *IntaRNA2* (2.1.0) [36] with parameters *--seedBP* 9, *--seedQRange* 1-9, and *--seedTRange* (*p*−5)*-*(*p*+3). As predefined energy parameter file, the file *turner99* from the *ViennaRNA* package (2.4.1) [37] was used. As described in [29], binding energies were obtained for six different substrate sequence window sizes (from 100 nt to 600 nt in 100 nt increments) and the averages and standard deviations were calculated. Therefore, these standard deviations do not reflect precision but robustness to the window size parameter in the calculation. Similar calculations were performed for the *mNf1* mRNA (NM_010897.2). The used threshold of −12 kcal/mol is a stronger binding free energy than the threshold of −4 kcal/mol used in [29], probably because the current study included the P1 extension, which provides additional stabilization.

### 2.2. Preparation of RNAs for in Vitro Experiments

Templates for the in vitro transcription of RNAs were prepared by PCR amplification of the corresponding ribozyme or substrate sequences from gBlocks (ordered from IDT DNA), or from plasmids that contained these gBlock sequences. The template for the 501-nucleotide long fragment of *mNf1* was generated by PCR amplification from a cloned *mNf1* fragment with a 5′-PCR primer that included the T7 promoter, a hammerhead ribozyme (italics) and the annealing region to the *mNf1* sequence (underlined) in 5′-AATTTAATACGACTCACTATA*GGGAAGAGATACTGA CGAGCTAAGCGAAACTGCGGAAACGCAGTC*TATCTCTTCCATGTTGTCACTTTC and 3′-PCR primer GATAATCTGCTTTATCTGCC. The 5′-hydroxyl group was generated by co-transcriptional cleavage of the RNA by the hammerhead ribozyme. The template to transcribe the 271-nucleotide long *mNf1* substrate was generated by PCR with a 5′ PCR primer AATTTAATACGACTCACTATAgagacc aagcaagttttgag and the 3′-PCR primer gagctagttctgtccactg. The template to transcribe the 1001-nucleotide long *mNf1* substrate was generated by PCR with a 5′ PCR primer AATTTAATACGACTCACTATAgaggcttagggtctatca and the 3′-PCR primer cttgtcattgaatat ccggag. The 5′-PCR primers add the promoter for T7 RNA polymerase is shown in uppercase.

In most cases, 2 nM plasmid was amplified by 22 PCR cycles to generate a clean, saturated band of PCR product as judged on ethidium bromide stained 1% agarose 1× TAE gels. The PCR product was purified on nucleospin PCR cleanup columns (Macherey-Nagel, Düren, Germany) and its concentration determined by its absorption at 260 nm. RNAs were generated by run-off transcription from PCR products by incubating 150 nM PCR product with 40 mM Tris/HCl pH 7.9, 2.5 mM spermidine, 26 mM MgCl_2_, 0.01% Triton X-100, 5 mM DTT, 2.5 mM of each NTP, and T7 RNA polymerase at 37 °C for two hours. Products were separated by 7 M urea 5% PAGE, visualized by UV shadowing, excised, and eluted by agitating for 2–6 h at 4 °C in 300 mM NaCl. After ethanol precipitation, pellets were washed with cold 75% ethanol to remove NaCl, which is inhibitory to the ribozyme. Purified RNAs were dissolved in water, and their concentration determined by UV spectroscopy. Purified *mNf1* mRNA fragments were 5′-radiolabeled using γ[^32^P] ATP and PNK (New England Biolabs, Ipswich, MA, USA) and purified by 7 M urea 5% PAGE as described above.

### 2.3. In Vitro Trans-Splicing Experiments

The substrate and ribozyme were dissolved separately in 1× reaction buffer (50 mM MOPS/KOH pH 7.0, 135 mM KCl, 5 mM MgCl_2_, 2 mM spermidine, 20 µM GTP) and incubated at 37 °C for 5 min to support folding of the ribozyme. Then the two solutions were combined for final concentration of 100 nM ribozyme and 5 nM template and incubated at 37 °C for 1 h if not indicated otherwise. The reactions were quenched by adding a 2-fold molar excess of EDTA over MgCl_2_, and processed by ethanol precipitation, resuspended in 40% formamide 1× TBE PAGE loading buffer, and heated to 80 °C for 2 min. After separation by 7 M urea 5% PAGE, gels were exposed to phosphorimager screens for 20 h. The screens were scanned in a Typhoon phosphorimager (Cytiva, Marlborough, MA, USA), and bands were quantified by the rectangle method using Quantity One (Bio-Rad, Hercules, CA, USA) and subtracting the background with rectangles placed in the same lane, near the band in an area without band. The data were processed and graphed using Microsoft Excel. Trans-splicing efficiencies were calculated as the fraction of trans-splicing product divided by the sum of trans-splicing product, cleavage product, and unreacted substrate.

### 2.4. Combinatorial Selection of Improved Extended Guide Sequences on NF1 mRNA

Combinatorial efforts to optimize the EGS are complicated by the fact that the EGS is not carried over into the splice products. Therefore, if a library of ribozymes with different EGSs would be used to splice a substrate, it would not be possible to identify the EGSs of the successful ribozymes based on the sequencing analysis of splice products. To alleviate this problem, barcodes were added to the ribozyme 3′-tails that would be visible in the sequences of the splicing products. By generating an EGS library with limited complexity (~100 million), attaching barcodes to the 3′-termini of this library, and sequencing this library (Figure S1), most EGSs in this library could be identified by the corresponding barcode in the splicing products.

Trans-splicing reactions in the combinatorial experiment used seven different incubation conditions (A–G): (A) The standard condition used 100 nM ribozyme library, 100 nM of the 501 nt long *mNf1* substrate, 50 mM MOPS/KOH pH7.0, 135 mM KCl, 2 mM spermidine, 5 mM MgCl_2_, 20 µM GTP with 5 min pre-incubation at 37 °C before combining ribozyme library and substrate. Condition (B) used 10 nM substrate, (C) used a shorter substrate (271 nt long), (D) used a longer substrate (1001 nt long), (E) used 1.5 mM MgCl_2_, (F) used 200 µM GTP, and (G) used a 1 h long pre-incubation (see also Figure S1). The splicing products were processed as described for the trans-splicing assay, reverse transcribed using Superscript III reverse transcriptase (Invitrogen, Waltham, MA, USA) and prepared for high throughput sequencing by PCR reactions that added Illumina adapters and Illumina indices (Illumina, San Diego, CA, USA).

High Throughput Sequencing (HTS) analysis of the splicing products was done separately for each of the seven reaction conditions. Forward reads were filtered for valid splicing products with the sequence CCTGGCTCC**T**ATGAACGCGA, then reverse reads were filtered for valid ribozyme 3′-tails with barcodes GTGGTGATCAGGCATGGANNNNNNNNNNNNNNNNNNNN. The barcodes were extracted and sorted by the number of occurrences. Using the sequencing results of the library that associated EGSs with barcodes, the EGSs that were uniquely identified by the corresponding barcodes were considered for subsequent testing. For each of the seven pools representing a different reaction condition, the EGSs associated with the five most frequent barcodes were chosen. A 6th EGS was added if a barcode had already been selected from a previous pool. Initially, a total of 35 selected EGSs were chosen for biochemical analysis (Table S1). However, 15 of the 35 EGSs resulted in poor transcription efficiency and were therefore dropped from the biochemical screen.

### 2.5. Ribozyme Expression Constructs

To express ribozymes in cells, gBlocks (IDT DNA) containing ribozymes and promoters were cloned into the expression plasmid pcDNA3.1/Zeo(+) or pCR2.1 between the SnaBI and XbaI sites in pcDNA3.1 for expression with the CMV promoter.

The ribozyme’s 3′ replacement sequence was designed to contain the first 30 nt of the WT *mNf1* sequence at the splice site (ATGAGAGGGAGACATTTGCTTTGACGTCCC), mimicking the repair sequence. To simplify the detection of splice product, we extended this sequence by a 50 nt long subsequence from the eGFP gene (TGGAGAGCGACGAGAGCGGCCTGCCCGCCATGGAGATCGAGTGC CGCATC).

Ribozyme constructs for U6-controlled expression were Topo-cloned into pCR2.1, which was chosen because it is devoid of promoters interfering with U6 in human cells. Because the U6 promoter drives transcription by the Pol III RNA polymerase, which terminates at sequences with multiple consecutive Ts (in general, 4 or more), several Ts were added at the 3′-terminus of the ribozyme sequence to terminate transcription. Additionally, the single occurrence of ‘TTTT’ on the ribozyme 3′ end was changed to ‘GTTT’. Because the U6 promoter requires the transcribed sequence to start with a G, such a G was added to the ribozyme’s 5′-terminus. In addition to the ribozyme variants with promising EGSs 22, 25, and 30, arbitrary EGSs were used as controls, as well as a ribozyme with no EGS (only IGS). Note that EGS25 could not be used with the U6 promoter because it contains T(4).

### 2.6. Expression of Ribozymes in HEK293 NF1−/− and NF1−/−; mNf1 Cells

Ribozymes were tested using HEK293 NF1−/− cells as well as HEK293 *NF1−/−* cells stable expressing wildtype his-tagged *mNf1* cDNA. Previously, the Wallis lab had generated *hNF1−/−* cells using CRISPR guides flanking *hNF1* exon 2, leading to the deletion of exon 2, thereby introducing a frameshift [38].

HEK293 *NF1−/−* cells were seeded at 7.5 × 10^5^ cells per well in a 6 well plate. The following day cells were transfected with 1 µg of *mNf1* cDNA (plasmid) and 3 µL of Lipo D (SignaGen laboratories, Frederick, MD, USA, Cat.SL100668) reagent for 24 h. The third day, media was replaced with fresh media without transfection reagents or plasmids. On the fourth day, selection began with 500 ug/mL G418 (RPI Corp., Mt. Prospect, IL, USA, Cat. G64500) for 14–21 days (changing medium every 3–4 days) until single cell colonies formed. Cells remained under continuous selection while they were picked and harvested. The picked cells were then grown in a 10 cm dish with selection media until confluent and frozen back.

Cells were transfected using 6 uL of LipoD293 (SignaGen) with 2 µg of plasmid ribozyme DNA (according to manufacturer’s protocol) per six-well dish. RNA was extracted 48 h post transfection using RNeasy (Qiagen, Hilden, Germany). Beta-mercaptoethanol (10 µL of 14.3 M B-mercapto to 1 mL RLT buffer) was added to decrease RNase activity (see also Qiagen’s RNeasy mini handbook). Most samples yielded RNA in excess of 15 µg. Reverse transcription used 1 µg of RNA and the iScript kit (Bio-Rad), following manufacturer’s instructions. As RT primer, we used GATGCGGCACTCGATCTC (R1).

Reverse transcription products were amplified with two sets of nested PCR primers, first F1 (ACAGAGTTCTCGTTACCCAAAT), which binds 115 bp upstream of the *mNf1* splice site, and R1 (GATGCGGCACTCGATCTC), then F2 (GCCTTCCGTTCCAGTTA, or TGGCATTAGCAAAGTCAAG) and R2 (GCTCTCGTCGCTCTCCA). RT-qPCR raw data are shown in Figure S2A, with threshold cycles converted to relative abundance in Figure S2B. PCR products were cloned, and 20 clones were sequenced.

## 3. Results

### 3.1. Computational Prediction of Efficient Splice Sites on NF1 mRNA

**Figure 2:**
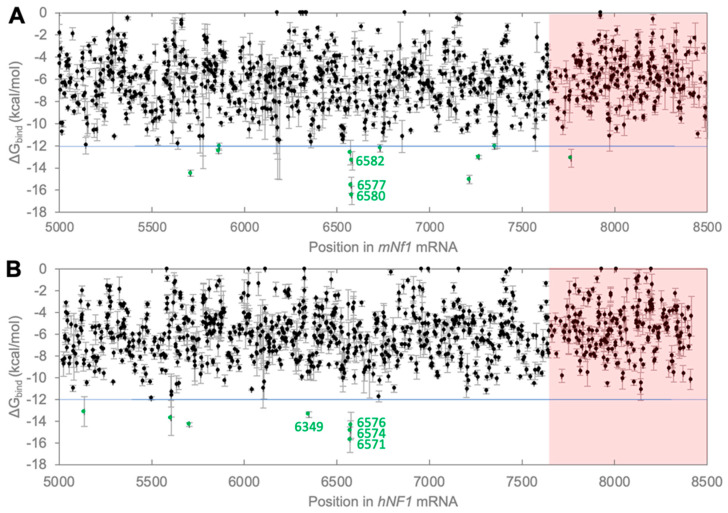
Computed substrate recognition free energy for all uridylates in the last 3500 nucleotides of (**A**) mouse *Nf1* mRNA and (**B**) human *NF1* mRNA. The free substrate recognition energy is plotted as a function of the nucleotide position, with each circle representing one uridylate. For each uridylate, six different window sizes were used for calculating the recognition free energies, thereby reporting on the robustness of each prediction. The circles show the average free energy, and error bars show the standard deviation between the six different window sizes. The threshold of −12 kcal/mol is indicated with a horizontal blue line. Uridylates with recognition free energies stronger than −12 kcal/mol are marked in green. The red area marks the sequence that would need to be replaced for repair of the mutation A7648T in *hNF1* mRNA, which corresponds to the clinically relevant patient-mutation R2550X (P21359-2, protein isoform 1, length 2818 aa).

To identify a suitable splice site for *NF1* repair, a computational approach was used. The only strict requirement for a splice site on the substrate RNA is a uridylate (U), therefore in principle, all 2308 uridylate residues on the 8454-nucleotide long *NF1* mRNA could serve as splice site [39,40,41]. However, most uridylates cannot mediate efficient trans-splicing due to their inaccessibility within the local substrate secondary structure, a weak P1 helix of the flanking nucleotides with the ribozyme’s IGS, and/or self-structure formation of the corresponding nucleotides in the ribozyme’s 5′-terminus. Together, these three factors are the key determinants of whether a splice site will be efficient or not, and the sum of their computed free energies is a good predictor of in vitro trans-splicing efficiency [29]. Given our long-term goals of developing trans-splicing ribozymes that work in mouse models as well as in humans, we computed the free energy of substrate recognition for both human (*hNF1*) and murine *NF1* (*mNf1*) transcripts for the last ~3500 nt of *mNf1* and *hNF1* mRNA (only the coding regions), respectively (Figure 2). Only the last ~3500 nt were used because this limits the size of the ribozyme’s ‘repair sequence’ in its 3′-tail to 3500 nucleotides, and keeps the size of the overall construct below 4000 nucleotides, which would be small enough for delivery by AAV vectors [42].

When computing the free substrate binding energies of the ribozyme to mouse and human *NF1* mRNA, the P1 extension (3 base pairs) was used in addition to the P1 helix (as in [29]) because this made the computational model more similar to the desired final product—which includes a P1 extension. This computation predicted several efficient splice sites that appear in both mRNAs (Figure 2). Twelve uridylates in *mNf1* mRNA and seven uridylates in *hNF1* mRNA had a binding free energy of −12 kcal/mol or better (green symbols in Figure 2A,B, below the blue line). These two sets of uridylates shared four positions (U5709/5703, U6577/6571, U6580/6574, and U6582/6576 in *mNf1* and *hNF1* mRNA, respectively). We focused on the latter three uridylates because they were clustered together and significantly closer to the target RNA’s 3′-end, implying a shorter replacement sequence. Also, all uridylates appear in a similar sequence context in both mRNAs so that trans-splicing could be optimized on *mNf1* mRNA and the same ribozyme used later on *hNF1* mRNA. In addition, we chose the nearby located uridylate (U6349/6355), which had an associated predicted binding free energy below the cut-off in *hNF1* (albeit not in *mNf1*). Moreover, because all four target sites are at, or before the uridylate 6582/6576, they can be used to repair any mutation that occurs downstream of this position. For example, they would allow the repair of the clinically relevant patient-mutation A7648T in *hNF1*, which corresponds to amino acid mutation R2550X in protein isoform 1 (P21359-2; transcript NM_000267.3, variant 2). This is indicated in Figure 2A,B as the portion of the plots highlighted in light red to emphasize that any uridylate in the white zone, but not in the red zone could be targeted to repair a mutation in position 7648, the location of the genetic mutation causative for p.R2550X. The four putative splice positions U6349/6355, U6577/6571, U6580/6574, and U6582/6576 in *mNf1* and *hNF1* mRNA, respectively, meet this criterion.

### 3.2. Experimental Validation of Efficient Splice Sites

**Figure 3:**
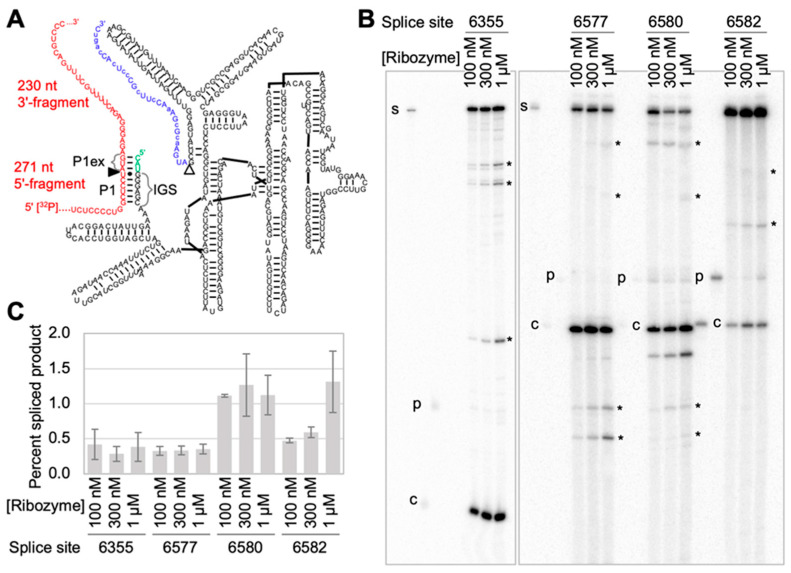
Experimental testing of four computationally identified splice sites on *mNf1* mRNA. (**A**) Secondary structure schematic of the ribozyme/substrate interactions. The 501 nucleotide (nt) long *mNf1* mRNA fragment (red) is recognized by the ribozymes via a 6 basepair P1 and a 3 basepair P1 extension helix (P1ex). In the shown example of recognition at splice site 6580 (filled triangle), substrate cleavage generates a 5′-fragment of 271 nt length, which contains the [^32^P] radiolabel. The ribozyme 3′-tail (blue) contains exactly 30 nucleotides, which are added to the substrate 5′-fragment to generate in this case a 301 nt long splicing product. The secondary structure of the ribozyme is as described in Figure 1. (**B**) Autoradiogram of trans-splicing products with 5′-[^32^P] radiolabeled *mNf1* mRNA, after separation of the samples by denaturing 5% polyacrylamide gel electrophoresis (PAGE). The bands of splicing substrate (s), cleavage product (c) and splicing product (p) are labeled. For each splice site, the corresponding ribozymes were used at three different concentrations as labeled on top of the images. Off-target cleavage/splicing products are labeled with asterisks. The *mNf1* substrate fragment used for splice site 6355 is different from the *mNf1* substrate at splice sites 6577, 6580, and 6582 to facilitate a clear separation of reaction products (see also Table S2). The uncropped autoradiogram are shown in Supplementary File S1. (**C**) Trans-splicing efficiencies as function of ribozyme concentration and splice sites at uridylates 6355, 6577, 6580, and 6582. Error bars are standard deviations from three independent experiments.

To test experimentally whether the four uridylates 6355, 6577, 6580, and 6582 would function as efficient splice sites on *mNf1* mRNA, four trans-splicing ribozyme constructs were generated to target these uridylates. They were tested in vitro because that allows easier control of experimental parameters and measurement of reaction efficiency, and because splice sites that were found efficient in vitro generally also performed well in cells [17,20,43,44,45]. For the in vitro experiments, the *mNf1* mRNA was truncated to 501 nt (splice sites 6577, 6580, 6582) or 524 nt (splice site 6355) to avoid technical difficulties with longer RNAs (Table S2). The truncations were chosen such that the region around the tested splice sites was predicted to fold similarly as the full-length *mNf1* mRNA (using *mFold* [46]), and to position the splice site such that splicing would be clearly visible in a gel shift (Figure 3). All four splice sites generated detectable amounts of splice product at 1 µM ribozyme concentration. Uridylates 6580 and 6582 resulted in the strongest signals for splice products. Uridylate 6580 gave a clear splice signal even at the low ribozyme concentration of 100 nM and was therefore chosen as the splice site for further optimization of the trans-splicing reaction.

### 3.3. Combinatorial Screen for EGSs That Mediate Efficient Splicing at Splice Site 6580 on NF1 mRNA

**Figure 4:**
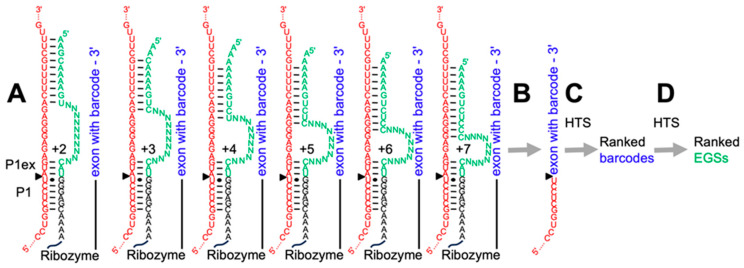
Experimental design for the identification of efficient EGSs for ribozymes targeting splice site 6580 on *mNf1* mRNA. (**A**) Secondary structure schematic showing the design of the six ribozyme sub-libraries that differed in the register between P1 extension (P1ex) and antisense duplex. The register (+2 to +7) is defined by the difference in length between the substrate side (red) and the EGS side (green) of the two strands between antisense duplex and P1ex. The ribozyme (black) and ribozyme 3′-tail with barcode (blue) are not shown as sequence for clarity. (**B**) After the splicing reaction, the barcodes appeared in the splicing products. (**C**) The reverse-transcribed and PCR-amplified splicing products were analyzed by High Throughput Sequencing (HTS) analysis and ranking the most enriched barcodes. (**D**) Identification of the EGSs that corresponded to the most enriched barcodes led to a ranked list of 20 promising EGSs.

At a given splice site, the splicing efficiency can be increased dramatically by attaching an extended guide sequence (EGS) to the ribozyme’s 5′-terminus [30]. Trans-splicing efficiency is increased by an antisense duplex between the 5′-terminal portion of the EGS and the substrate, and an incompletely paired structure between this antisense duplex and the P1 extension. However, it is currently unclear what is the best sequence in the EGS between this antisense duplex and the P1 extension, and in which register the antisense duplex should be with respect to the P1 extension [32,33]. To determine sequences in this EGS region that were optimally adjusted to the local sequence environment of splice site 6580 on *mNf1* mRNA, we generated a combinatorial library of trans-splicing ribozymes with an IGS that targeted them to *mNf1* splice site 6580 and contained a 3-base pair P1 extension, a 10-nucleotide long randomized sequence in the center of the EGS, and 8–10 nucleotides designed to form an antisense duplex with the *mNf1* substrate (Figure 4A). The difference between 8 and 10 base pairs arose because one or two As are added for more efficient transcription. The antisense duplex was placed into six different registers between the P1 duplex and the antisense duplex.

The ribozyme library was incubated with the described 501 nt long fragment of *mNf1* mRNA under trans-splicing conditions, generating splicing products that contained the 5′-portion of the *mNf1* mRNA fragment and the 3′-exon of the ribozyme with barcode (Figure 4B). This incubation was done under seven different reaction conditions to identify ribozyme variants that were robust to different reaction conditions. The splicing products were reverse transcribed with a primer complementary to the ribozyme 3′-tail, and PCR amplified by PCR primers binding to the *mNf1* mRNA 5′-fragment and the ribozyme 3′-tail. The barcodes in the successful splicing products were identified by High Throughput Sequencing (HTS) analysis of the splicing products (Figure 4C). The EGSs associated with the most highly ranked barcodes were then identified by HTS of the original library, resulting in 20 promising EGSs (Figure 4D, Table 1).

### 3.4. Experimental Testing of EGSs for Ribozyme 6580

**Figure 5:**
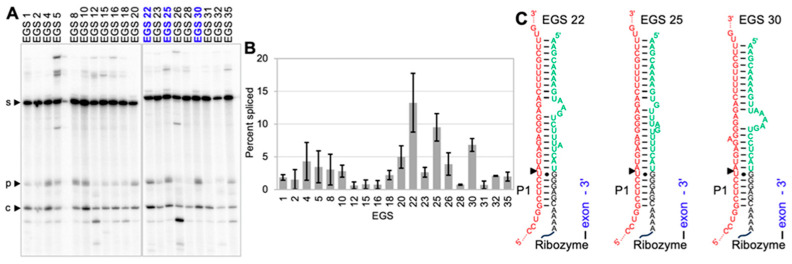
In vitro analysis of 20 EGSs for their benefit on trans-splicing at *mNf1* mRNA. (**A**) Autoradiogram of ribozyme trans-splicing products with a 5′-[^32^P] radiolabeled *mNf1* mRNA fragment after denaturing 5% polyacrylamide gel separation. The EGSs specific for each ribozyme are given on top. Bands of substrate (s), splice product (p), and cleavage product (c) are indicated. The uncropped blots are shown in Supplementary File S1. (**B**) Trans-splicing efficiency as percent of *mNf1* mRNA converted to splicing product. Error bars are standard deviations of three independent experiments. (**C**) Secondary structures that are predicted to be formed by the three most efficient EGSs. Color coding is as in Figure 1.

Ribozyme 6580 variants with 20 different EGSs identified above were subjected to biochemical analysis. To do this, 100 nM of each ribozyme variant were reacted with 5 nM of 5′-[^32^P]-radiolabeled *mNf1* substrate for one hour under near-physiological conditions, the reaction products were separated by denaturing polyacrylamide gel electrophoresis and quantified by phosphor imaging (Figure 5A) in three independent experiments (*n* = 3). The most efficient EGSs were EGS22, EGS25, and EGS 30 (Figure 5B). The overlapping error bars indicate that EGS 22 and EGS 25 are not significantly different in their trans-splicing activity, while the non-overlapping error bars suggest that EGS 30 is significantly less efficient than EGS 22. The three EGSs form very compact structures with the target site, based on secondary structure predictions (Figure 5C). The observation that all unpaired nucleotides were purines (A and G) can be explained by the pyrimidines (U, C) being paired to this purine-rich stretch of the substrate. The three EGSs 22, 25, and 30 were chosen for analysis in human cells.

### 3.5. Trans-Splicing in HEK293 Cells

To test the trans-splicing ability of the developed ribozyme variants in human cells, it was necessary to address three aspects: what is the best RNA target in HEK293 cells, what is the most suitable promoter for ribozyme expression, and which sequences should be trans-spliced by the ribozyme for unambiguous analysis. First, mouse *mNf1* was chosen as target to allow later testing of ribozymes in *NF1*^mut^ mouse models. Previously, the Wallis lab had developed a HEK293 *NF1−/−* cell line, which lacks neurofibromin protein due to a frameshift mutation, resulting in a premature stop codon [38]. These cells were transfected to stably express wildtype (WT) *mNf1* cDNA, resulting in the *hNF1−/−; mNf1* genotype. However, HEK293 *NF1−/−* cells are still producing mutant *hNF1* mRNA containing frameshifts, which may interfere with ribozyme splicing the intended target *mNf1*. The former would not produce neurofibromin, even if trans-spliced. A comparison between the ribozyme binding sites on *mNf1* and *hNF1* RNA suggested that the ribozymes are unlikely to discriminate between these two RNAs (Figure S3), and therefore the ratio in the splicing products between *mNf1* and *hNF1* RNA would largely depend on their expression levels in the cells.

The most suitable promoter for ribozyme expression would generate enough ribozyme for detectable trans-splicing but not so much that it would interfere with functions of the cell. Two promoters were tested in this study, the CMV promoter and the U6 promoter. Ribozyme variants with EGS 22, 25, and 30 were expressed in HEK293 *hNF1−/−; mNf1*+ cells, and the expression levels were quantitated by RT-qPCR (Figure S2). The ribozymes did not carry the complete *mNf1* replacement sequence on their 3′-ends, but rather a construct consisting of 30 nucleotides following the splice site on *mNf1* (positions 6581-6610), followed by a 50 nt long barcode that allowed discerning splice products from substrate sequences. The RT-qPCR results showed that all ribozymes were detectable in the cells by RT-qPCR, with the CMV promoter leading to about 30-fold higher ribozyme expression than the U6 promoter, consistent with the known strength of these promoters [47,48]. Expression of the ribozyme with the U6 promoter generated ribozyme levels similar to the level of hypoxanthine phosphoribosyltransferase mRNA (*HPRT*), suggesting a copy number of about 1–30 ribozyme molecules per cell [49]. Given the ~30-fold higher expression level with the CMV promoter, about 30–900 ribozyme molecules per cell were generated when ribozyme expression was driven by the CMV promoter.

**Figure 6:**
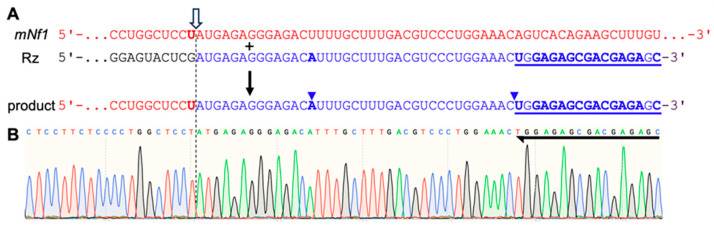
Sequence of a representative trans-splicing product isolated from HEK293 cells. (**A**) Sequence of the mouse *Nf1* mRNA (*mNf1*; red) with the splice site U in bold, the ribozyme (black) with its 3′-tail (blue), and the sequence of the product isolated from HEK293 cells (red 5′-portion and blue 3′-portion). The outlined white arrow, and the vertical dashed line indicate the position of the splice site. The ‘+’ sign and the vertical black arrow indicate that the *mNf1* sequence and the ribozyme 3′-tail are recombined to form the product sequence. (**B**) Chromatogram of the product sequence after RNA isolation from HEK293 cells, reverse transcription, PCR and cloning. The chromatogram is aligned with the sequences in (**A**). The black arrow on the right indicates the primer annealing site for the nested (inner) PCR amplification of reverse transcribed products. The left blue arrowhead points to a mutation introduced by ribozyme splicing, confirming the sequence as splicing product. The right blue arrowhead indicates the correct position of the primer binding site, confirming that this site was not added by erroneous priming but by ribozyme-based splicing. The sequence of the cloned DNA is indicated above the chromatogram.

To test the different combinations of ribozyme EGS and promoter, HEK293 *hNF1−/−* and HEK293 *hNF1−/−; mNf1*+ cells were transiently transfected with the ribozyme expression constructs. Cellular RNA was isolated and RNAs containing the ribozyme’s 3′-tail were reverse transcribed. qPCR results suggested that the ribozyme with EGS30 and controlled by a U6 promoter produced the most trans-splicing products (Figure S2). To test whether the U6-EGS30 trans-splicing ribozyme generated the correct splicing product, the cDNA of trans-splicing products was cloned and sequenced. The differences between ribozyme 3′-tail and substrate 3′-portion allowed the unambiguous identification of splicing products as opposed to substrate sequences. Out of 20 sequences, 15 showed the correct splice product sequence (Figure 6), while one additional sequence showed trans-splicing but with 4 mismatches (presumably due to errors in transcription or reverse transcription). The ribozyme 3′-tail contained a U -> A mutation that also appeared in the isolated sequences 14 nucleotides downstream of the splice site. Additionally, the 3′-PCR primer binding site (underlined in Figure 6A) was precisely in the right location, which would not be the case if the RT and 3′-primers had randomly annealed to an unspliced *mNf1* RNA. These two observations confirmed that the ribozymes successfully trans-spliced *mNf1* mRNA in HEK293 cells. Additionally, the splice junction (red GGCTCCT-ATGAGA blue) was intact in all 15 sequences, without frameshift or mutation, showing that this ribozyme can generate splicing products that could be translated into the correct protein sequence.

## 4. Discussion

This study showed the development of trans-splicing group I intron ribozymes that can be used to repair mutated *NF1* mRNAs in cells. After identifying and experimentally validating suitable splice sites shared by *mNf1* and *hNF1* mRNA, the ribozyme’s extended guide sequence (EGS) was optimized in a combinatorial approach and the most promising EGSs were individually confirmed in vitro. After transfection of ribozyme expression constructs into HEK293 cells, the ribozyme’s correct splicing products were identified.

The trans-splicing efficiency of the developed ribozymes in cells is currently unclear. Initial RT-qPCR experiments to quantify the trans-splicing efficiency in HEK293 cells suffered from high background with unrelated RNAs, and their 4-fold stronger signal in the samples with ribozyme could therefore only be used to qualitatively confirm that trans-splicing was indeed taking place. Future efforts will optimize the ribozyme 3′-tail sequence to minimize background effects and thereby obtain more quantitative results.

While two different promoters were employed (CMV and U6), the promoter leading to higher expression level (CMV) is not necessarily more suitable: Transcription by the CMV promoter requires a 5′-terminal leader sequence [50] that may interfere with the ribozyme’s ability to trans-splice. In contrast, the U6 promoter (which is used in biology to drive the expression of short RNAs) does not require such an added 5′-sequence [51], and could therefore be the more suitable promoter for the in-cell expression of therapeutic ribozymes.

It is currently unknown how much functional neurofibromin is required to prevent or treat different pathologies associated with NF1, which may vary for different cell types and/or microenvironments. It has been shown that mice with one null *NF1* allele and one G848R allele express about 25% wild type neurofibromin levels and do not have overt phenotypes, indicating that 25% expression is sufficient in mice [52]. Hence, as a tumor suppressor gene that stimulates Ras-GTPase, we expect that a threshold of 10–15% restoration of neurofibromin will be therapeutic for many associated pathologies.

The trans-splicing ribozymes described here were foremost selected for trans-splicing efficiency but did not directly select against off-target effects. However, the used combinatorial procedure filters for the correct splicing products during the stage of high throughput sequencing analysis. Since the ribozymes are single-turnover ribozymes, any EGS that would mediate low specificity would likely lead to the loss of the corresponding ribozyme because it would be much more likely to splice at the more than 100 uridylates in the NF1 target RNA than at the correct splice site. Moreover, we do not expect that a low fraction of trans-splicing ribozymes with the NF1 mRNA’s 3′-portion would generate significant problems for the cell/ patient. Most likely, such off-target splicing products would result in ribosomal stalling and degradation of the specific mRNA molecule. Nevertheless, future efforts will be directed towards specifically quantifying and, if necessary, decreasing off-target splicing.

While our results provide a proof-of-concept for ribozyme-based *mNf1* and *hNF1* trans-splicing, trans-splicing efficiencies will have to be improved further for this strategy to become a viable therapeutic approach. We identified several directions for improving trans-splicing efficiencies and testing in cells. For instance, we only tested four splice sites in *mNf1* RNA. While those were among the most promising according to computational predictions, a trans-tagging experiment that tests splice sites competitively [53] could either confirm those splice sites as most optimal or identify even better splice sites. We anticipate that future experiments can co-optimize promoters, ribozyme variants, repair sequences, and delivery methods to make trans-splicing ribozymes useful for the therapy of Neurofibromatosis Type I.

## 5. Conclusions

This study showed that *NF1* mRNA harboring pathogenic variants can be targeted for repair by trans-splicing group I intron ribozymes. Using a computational approach, four splice sites were identified and validated in vitro. With a combinatorial approach, extended guide sequences were identified that increased trans-splicing efficiency. Expression of the top three ribozymes resulted in correct trans-splicing products in HEK293 cells. Overall, this study shows that trans-splicing group I intron ribozymes could be useful as a therapeutic approach to treat Neurofibromatosis Type I (NF1).

## Figures and Tables

**Table 1:** Sequences and biochemical activity of EGSs that were tested by in vitro trans-splicing on mNf1 mRNA. The EGSs were sorted based on their trans-splicing activity as shown in Figure 5B, with highest activity on top. On the DNA level, all constructs were preceded by the phi 2.5 promoter for T7 RNA polymerase 5′-AATTTAATACGACTCACTATT that allows transcription to start with an A, such that all transcripts started with 5′-AAG or 5′-AAC. The underlined sequence immediately at the 5′-terminus forms the antisense duplex with the substrate (compare to Figure 1). The underlined sequence on the right forms the P1 extension (CAT) and the IGS (GGGAGC) in one continuous duplex. The sequence between the antisense duplex and P1 extension (not underlined) stems from the randomized part of the library and is different for each EGS. The read count is the total number of reads corresponding to the specific EGS. Column 4 indicates the highest rank across the seven replicates. The two right columns show the average in vitro splicing efficiency and their standard deviation from three independent experiments (compare to Figure 5).

Name	Sequence	Read Count	Highest Rank	% Spliced	Stdev
EGS 22	5′-AAGCAAAAGTAAGTCTTTATCAT**G**GGAGC	7	3	8.2	5.3
EGS 25	5′-AAGCAAAAGTGTTTAGTTTTCAT**G**GGAGC	6 + 6	4 + 5	6.4	3.4
EGS 30	5′-AAGCAAAAGTTAAAAGTCCTCAT**G**GGAGC	5	5	5.4	3.4
EGS 20	5′-AAGCAAAAGTACAACTGGCCCAT**G**GGAGC	4	5	4.0	3.1
EGS 4	5′-AACAAAAGTCATGACAGCACCAT**G**GGAGC	4	4	3.9	4.0
EGS 8	5′-AAGCAAAAGTACATTACGCCCAT**G**GGAGC	8	3	3.1	3.6
EGS 26	5′-AAGCAAAAGTTGGGGGGGACCAT**G**GGAGC	6	1	2.1	2.1
EGS 23	5′-AAGCAAAAGTGTGTACCCGGCAT**G**GGAGC	7	4	2.1	1.5
EGS 10	5′-AACAAAAGTCAGTTAAGCTACAT**G**GGAGC	8	5	1.8	1.7
EGS 18	5′-AACAAAAGTCCCCCCAGGGACAT**G**GGAGC	4	3	1.8	1.0
EGS 5	5′-AACAAAAGTCTGTGCGTGGACAT**G**GGAGC	4	5	1.8	2.2
EGS 1	5′-AACAAAAGTCTACATACCTTCAT**G**GGAGC	5	1	1.5	1.0
EGS 35	5′-AAGCAAAAGTGCCAGGGCACCAT**G**GGAGC	32	5	1.5	1.4
EGS 32	5′-AACAAAAGTCAATGTTGGGCCAT**G**GGAGC	50	2	1.4	1.2
EGS 2	5′-AAGCAAAAGTGGGCCTCAACCAT**G**GGAGC	5	2	0.8	0.8
EGS 31	5′-AAGCAAAAGTGCTCTCCCCGCAT**G**GGAGC	51	1	0.6	0.7
EGS 28	5′-AACAAAAGTCCAAAGTGCTGCAT**G**GGAGC	5	3	0.5	0.4
EGS 16	5′-AAGCAAAAGTCCCCCAATTTCAT**G**GGAGC	4	1	0.5	0.6
EGS 15	5′-AAGCAAAAGTGCCGTAGCGTCAT**G**GGAGC	3	5	0.4	0.6
EGS 12	5′-AACAAAAGTCTCTCGGAGTCCAT**G**GGAGC	4	2	0.4	0.5

## Data Availability

All data used and analyzed during the current study are available from the corresponding authors upon reasonable request.

## Supplementary Materials

The following supporting information can be downloaded at: https://www.mdpi.com/article/10.3390/cancers17172749/s1, Figure S1: EGS library; Figure S2: RT-qPCR quantitation of ribozyme expression levels in HEK293 cells; Figure S3: Comparison of mNf1 (bottom) and hNF1 (top) DNA sequences around the splice site; Table S1: List of PCR primers that add 35 different EGSs; Table S2: Sequences of RNA constructs and PCR primers for in vitro reactions; File S1: Full pictures of the blots in Figure 3B and Figure 5A.

## Abbreviations

CMV	Cytomegalovirus.
EGS	Extended Guide Sequence.
HEK	Human Embryonic Kidney.
HTS	High Throughput Sequencing.
IGS	Internal Guide Sequence.
IND	Investigational New Drug.
NF1	Neurofibromatosis Type I.
P1ex	P1 extension.
