# Supplementary material for: Repair of Mutated NF1 mRNA with Trans-Splicing Group I Intron Ribozymes
Source: Cancers (Basel). 2025 Aug 23;17(17):2749. doi: 10.3390/cancers17172749 (PMC12427287; doi:10.3390/cancers17172749)
Supplement: Supplementary file 1 [file cancers-17-02749-s001.zip › Figure S1. EGSlibrary.pptx]

## Slide 1
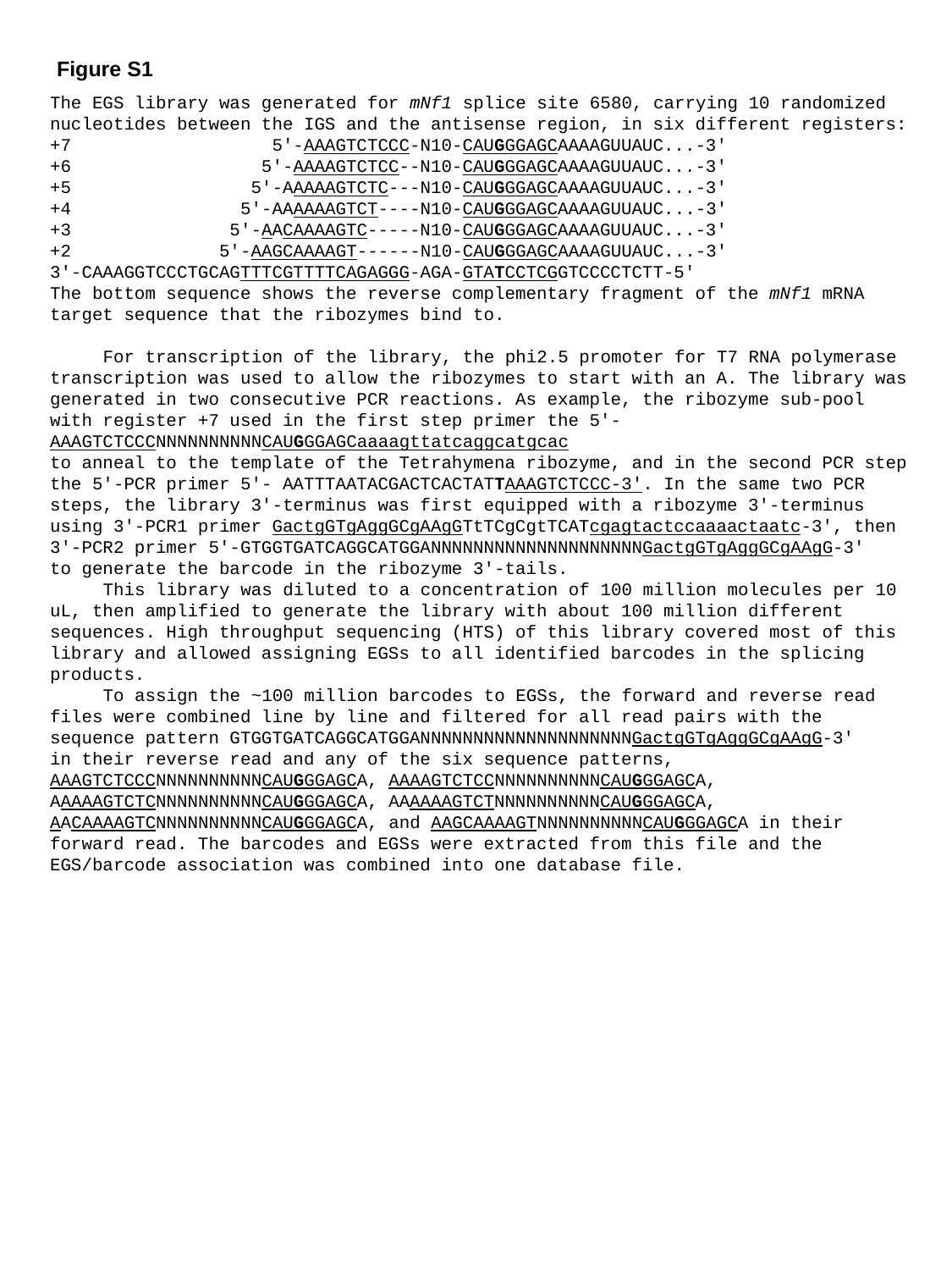

Figure S1
The EGS library was generated for mNf1 splice site 6580, carrying 10 randomized nucleotides between the IGS and the antisense region, in six different registers:
+7 5'-AAAGTCTCCC-N10-CAUGGGAGCAAAAGUUAUC...-3'
+6 5'-AAAAGTCTCC--N10-CAUGGGAGCAAAAGUUAUC...-3'
+5 5'-AAAAAGTCTC---N10-CAUGGGAGCAAAAGUUAUC...-3'
+4 5'-AAAAAAGTCT----N10-CAUGGGAGCAAAAGUUAUC...-3'
+3 5'-AACAAAAGTC-----N10-CAUGGGAGCAAAAGUUAUC...-3'
+2 5'-AAGCAAAAGT------N10-CAUGGGAGCAAAAGUUAUC...-3'
3'-CAAAGGTCCCTGCAGTTTCGTTTTCAGAGGG-AGA-GTATCCTCGGTCCCCTCTT-5'
The bottom sequence shows the reverse complementary fragment of the mNf1 mRNA target sequence that the ribozymes bind to.
 For transcription of the library, the phi2.5 promoter for T7 RNA polymerase transcription was used to allow the ribozymes to start with an A. The library was generated in two consecutive PCR reactions. As example, the ribozyme sub-pool with register +7 used in the first step primer the 5'- AAAGTCTCCCNNNNNNNNNNCAUGGGAGCaaaagttatcaggcatgcac
to anneal to the template of the Tetrahymena ribozyme, and in the second PCR step the 5'-PCR primer 5'- AATTTAATACGACTCACTATTAAAGTCTCCC-3'. In the same two PCR steps, the library 3'-terminus was first equipped with a ribozyme 3'-terminus using 3'-PCR1 primer GactgGTgAggGCgAAgGTtTCgCgtTCATcgagtactccaaaactaatc-3', then 3'-PCR2 primer 5'-GTGGTGATCAGGCATGGANNNNNNNNNNNNNNNNNNNNGactgGTgAggGCgAAgG-3'
to generate the barcode in the ribozyme 3'-tails.
 This library was diluted to a concentration of 100 million molecules per 10 uL, then amplified to generate the library with about 100 million different sequences. High throughput sequencing (HTS) of this library covered most of this library and allowed assigning EGSs to all identified barcodes in the splicing products.
 To assign the ~100 million barcodes to EGSs, the forward and reverse read files were combined line by line and filtered for all read pairs with the sequence pattern GTGGTGATCAGGCATGGANNNNNNNNNNNNNNNNNNNNGactgGTgAggGCgAAgG-3'
in their reverse read and any of the six sequence patterns, AAAGTCTCCCNNNNNNNNNNCAUGGGAGCA, AAAAGTCTCCNNNNNNNNNNCAUGGGAGCA, AAAAAGTCTCNNNNNNNNNNCAUGGGAGCA, AAAAAAGTCTNNNNNNNNNNCAUGGGAGCA, AACAAAAGTCNNNNNNNNNNCAUGGGAGCA, and AAGCAAAAGTNNNNNNNNNNCAUGGGAGCA in their forward read. The barcodes and EGSs were extracted from this file and the EGS/barcode association was combined into one database file.

## Slide 2
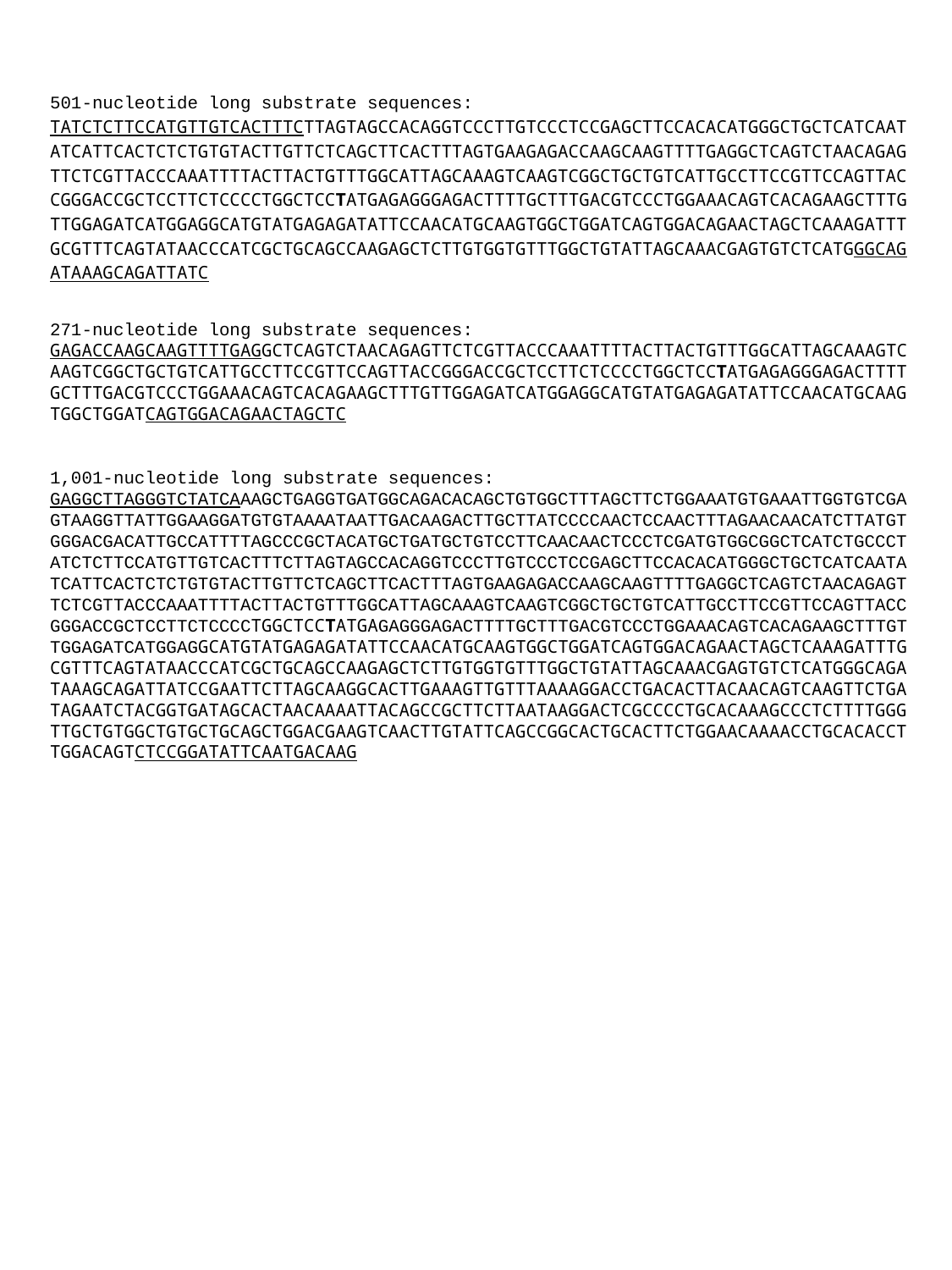

501-nucleotide long substrate sequences:
TATCTCTTCCATGTTGTCACTTTCTTAGTAGCCACAGGTCCCTTGTCCCTCCGAGCTTCCACACATGGGCTGCTCATCAATATCATTCACTCTCTGTGTACTTGTTCTCAGCTTCACTTTAGTGAAGAGACCAAGCAAGTTTTGAGGCTCAGTCTAACAGAGTTCTCGTTACCCAAATTTTACTTACTGTTTGGCATTAGCAAAGTCAAGTCGGCTGCTGTCATTGCCTTCCGTTCCAGTTACCGGGACCGCTCCTTCTCCCCTGGCTCCTATGAGAGGGAGACTTTTGCTTTGACGTCCCTGGAAACAGTCACAGAAGCTTTGTTGGAGATCATGGAGGCATGTATGAGAGATATTCCAACATGCAAGTGGCTGGATCAGTGGACAGAACTAGCTCAAAGATTTGCGTTTCAGTATAACCCATCGCTGCAGCCAAGAGCTCTTGTGGTGTTTGGCTGTATTAGCAAACGAGTGTCTCATGGGCAGATAAAGCAGATTATC
271-nucleotide long substrate sequences:
GAGACCAAGCAAGTTTTGAGGCTCAGTCTAACAGAGTTCTCGTTACCCAAATTTTACTTACTGTTTGGCATTAGCAAAGTCAAGTCGGCTGCTGTCATTGCCTTCCGTTCCAGTTACCGGGACCGCTCCTTCTCCCCTGGCTCCTATGAGAGGGAGACTTTTGCTTTGACGTCCCTGGAAACAGTCACAGAAGCTTTGTTGGAGATCATGGAGGCATGTATGAGAGATATTCCAACATGCAAGTGGCTGGATCAGTGGACAGAACTAGCTC
1,001-nucleotide long substrate sequences:
GAGGCTTAGGGTCTATCAAAGCTGAGGTGATGGCAGACACAGCTGTGGCTTTAGCTTCTGGAAATGTGAAATTGGTGTCGAGTAAGGTTATTGGAAGGATGTGTAAAATAATTGACAAGACTTGCTTATCCCCAACTCCAACTTTAGAACAACATCTTATGTGGGACGACATTGCCATTTTAGCCCGCTACATGCTGATGCTGTCCTTCAACAACTCCCTCGATGTGGCGGCTCATCTGCCCTATCTCTTCCATGTTGTCACTTTCTTAGTAGCCACAGGTCCCTTGTCCCTCCGAGCTTCCACACATGGGCTGCTCATCAATATCATTCACTCTCTGTGTACTTGTTCTCAGCTTCACTTTAGTGAAGAGACCAAGCAAGTTTTGAGGCTCAGTCTAACAGAGTTCTCGTTACCCAAATTTTACTTACTGTTTGGCATTAGCAAAGTCAAGTCGGCTGCTGTCATTGCCTTCCGTTCCAGTTACCGGGACCGCTCCTTCTCCCCTGGCTCCTATGAGAGGGAGACTTTTGCTTTGACGTCCCTGGAAACAGTCACAGAAGCTTTGTTGGAGATCATGGAGGCATGTATGAGAGATATTCCAACATGCAAGTGGCTGGATCAGTGGACAGAACTAGCTCAAAGATTTGCGTTTCAGTATAACCCATCGCTGCAGCCAAGAGCTCTTGTGGTGTTTGGCTGTATTAGCAAACGAGTGTCTCATGGGCAGATAAAGCAGATTATCCGAATTCTTAGCAAGGCACTTGAAAGTTGTTTAAAAGGACCTGACACTTACAACAGTCAAGTTCTGATAGAATCTACGGTGATAGCACTAACAAAATTACAGCCGCTTCTTAATAAGGACTCGCCCCTGCACAAAGCCCTCTTTTGGGTTGCTGTGGCTGTGCTGCAGCTGGACGAAGTCAACTTGTATTCAGCCGGCACTGCACTTCTGGAACAAAACCTGCACACCTTGGACAGTCTCCGGATATTCAATGACAAG
