# Supplementary material for: Repair of Mutated NF1 mRNA with Trans-Splicing Group I Intron Ribozymes
Source: Cancers (Basel). 2025 Aug 23;17(17):2749. doi: 10.3390/cancers17172749 (PMC12427287; doi:10.3390/cancers17172749)
Supplement: Supplementary file 1 [file cancers-17-02749-s001.zip › Figure S2. RzLevelInCellsRTqPCR.pptx]

## Slide 1
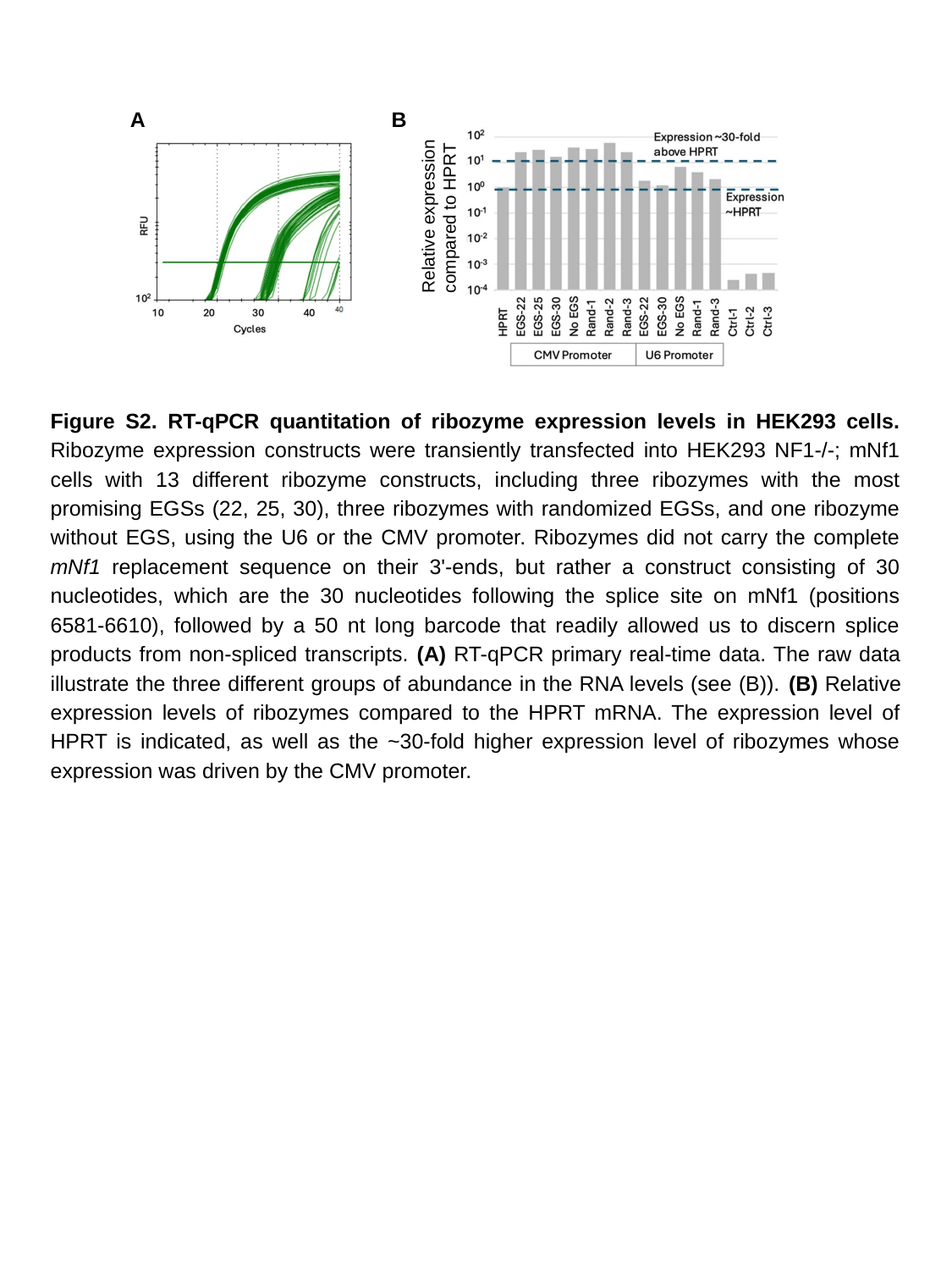

Relative expression
compared to HPRT
B
A
Figure S2. RT-qPCR quantitation of ribozyme expression levels in HEK293 cells. Ribozyme expression constructs were transiently transfected into HEK293 NF1-/-; mNf1 cells with 13 different ribozyme constructs, including three ribozymes with the most promising EGSs (22, 25, 30), three ribozymes with randomized EGSs, and one ribozyme without EGS, using the U6 or the CMV promoter. Ribozymes did not carry the complete mNf1 replacement sequence on their 3'-ends, but rather a construct consisting of 30 nucleotides, which are the 30 nucleotides following the splice site on mNf1 (positions 6581-6610), followed by a 50 nt long barcode that readily allowed us to discern splice products from non-spliced transcripts. (A) RT-qPCR primary real-time data. The raw data illustrate the three different groups of abundance in the RNA levels (see (B)). (B) Relative expression levels of ribozymes compared to the HPRT mRNA. The expression level of HPRT is indicated, as well as the ~30-fold higher expression level of ribozymes whose expression was driven by the CMV promoter.
