# Supplementary material for: Repair of Mutated NF1 mRNA with Trans-Splicing Group I Intron Ribozymes
Source: Cancers (Basel). 2025 Aug 23;17(17):2749. doi: 10.3390/cancers17172749 (PMC12427287; doi:10.3390/cancers17172749)
Supplement: Supplementary file 1 [file cancers-17-02749-s001.zip › Figure S3. CompareTargetsInHEK293Cells.pptx]

## Slide 1
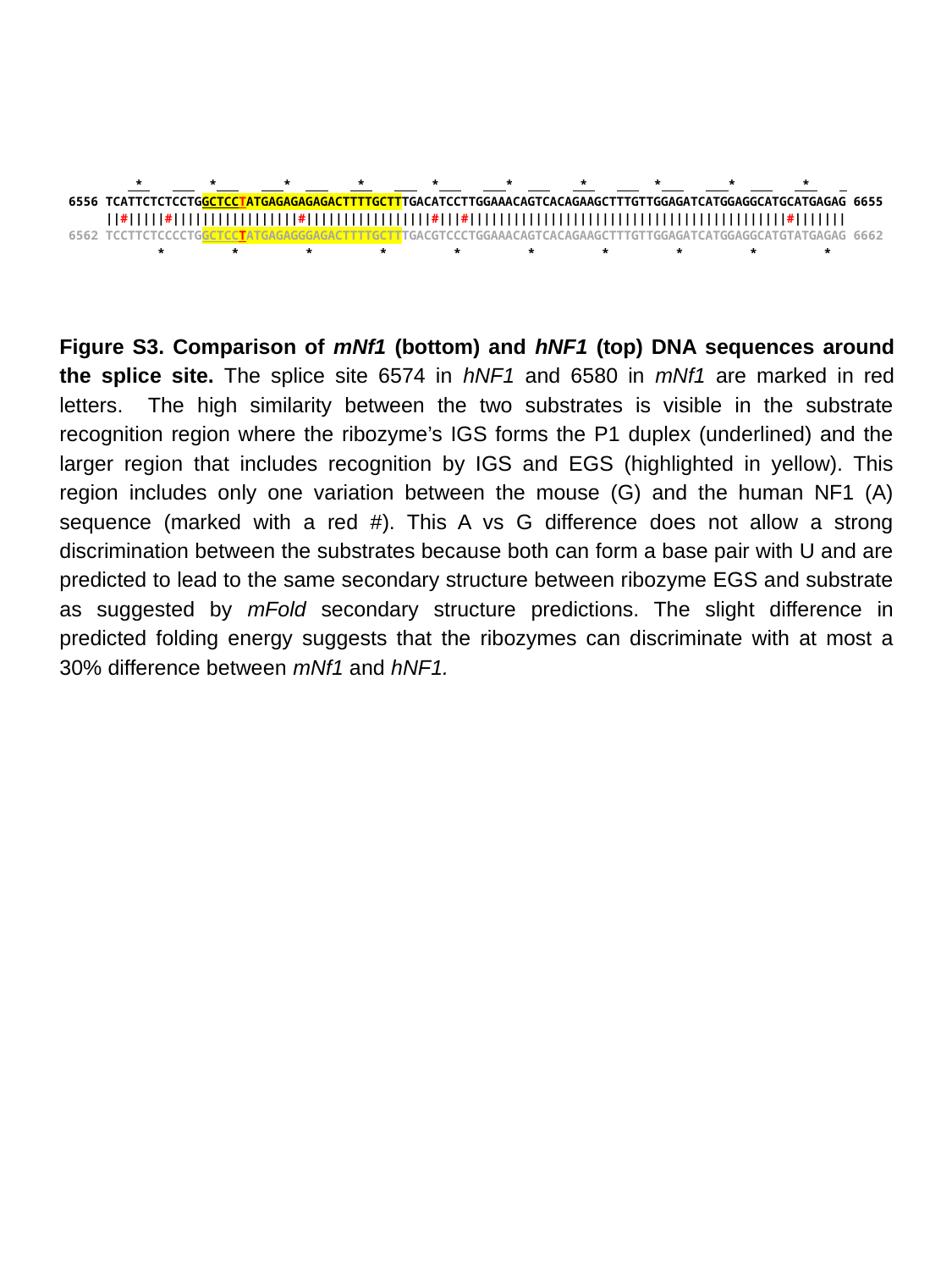

*         *         *         *         *         *         *         *         *         *      665
6556 TCATTCTCTCCTGGCTCCTATGAGAGAGAGACTTTTGCTTTGACATCCTTGGAAACAGTCACAGAAGCTTTGTTGGAGATCATGGAGGCATGCATGAGAG 6655
 ||#|||||#|||||||||||||||||#|||||||||||||||||#|||#|||||||||||||||||||||||||||||||||||||||||||#|||||||
6562 TCCTTCTCCCCTGGCTCCTATGAGAGGGAGACTTTTGCTTTGACGTCCCTGGAAACAGTCACAGAAGCTTTGTTGGAGATCATGGAGGCATGTATGAGAG 6662
            *         *         *         *         *         *         *         *         *         *
Figure S3. Comparison of mNf1 (bottom) and hNF1 (top) DNA sequences around the splice site. The splice site 6574 in hNF1 and 6580 in mNf1 are marked in red letters. The high similarity between the two substrates is visible in the substrate recognition region where the ribozyme’s IGS forms the P1 duplex (underlined) and the larger region that includes recognition by IGS and EGS (highlighted in yellow). This region includes only one variation between the mouse (G) and the human NF1 (A) sequence (marked with a red #). This A vs G difference does not allow a strong discrimination between the substrates because both can form a base pair with U and are predicted to lead to the same secondary structure between ribozyme EGS and substrate as suggested by mFold secondary structure predictions. The slight difference in predicted folding energy suggests that the ribozymes can discriminate with at most a 30% difference between mNf1 and hNF1.
