# Supplementary material for: Repair of Mutated NF1 mRNA with Trans-Splicing Group I Intron Ribozymes
Source: Cancers (Basel). 2025 Aug 23;17(17):2749. doi: 10.3390/cancers17172749 (PMC12427287; doi:10.3390/cancers17172749)
Supplement: Supplementary file 1 [file cancers-17-02749-s001.zip › Table S1. EGSPrimerSequences.pdf]

Table S1. List of PCR primers that add 35 different EGSs.

EGS01 5'-AATTTAATACGACTCACTATTAACAAAAGTCTACATACCTTCAT**G**GGAGCaaaagttatc-3'
EGS02 5'-AATTTAATACGACTCACTATTAAGCAAAAGTGGGCCTCAACCAT**G**GGAGCaaaagttatc-3'
EGS03 5'-AATTTAATACGACTCACTATTAAAAAAGTCTTATTTAGATTCAT**G**GGAGCaaaagttatc-3'
EGS04 5'-AATTTAATACGACTCACTATTAACAAAAGTCATGACAGCACCAT**G**GGAGCaaaagttatc-3'
EGS05 5'-AATTTAATACGACTCACTATTAACAAAAGTCTGTGCGTGGACAT**G**GGAGCaaaagttatc-3'
EGS06 5'-AATTTAATACGACTCACTATTAAAAGTCTCCCTCGTAGAGCCAT**G**GGAGCaaaagttatc-3'
EGS07 5'-AATTTAATACGACTCACTATTAAAAAGTCTCGAGAACGGAGCAT**G**GGAGCaaaagttatc-3'
EGS08 5'-AATTTAATACGACTCACTATTAAGCAAAAGTACATTACGCCCAT**G**GGAGCaaaagttatc-3'
EGS09 5'-AATTTAATACGACTCACTATTAAAAAAGTCTATAGCACGCGCAT**G**GGAGCaaaagttatc-3'
EGS10 5'-AATTTAATACGACTCACTATTAACAAAAGTCAGTTAAGCTACAT**G**GGAGCaaaagttatc-3'
EGS11 5'-AATTTAATACGACTCACTATTAAAAAAGTCTTATTAGTGGGCAT**G**GGAGCaaaagttatc-3'
EGS12 5'-AATTTAATACGACTCACTATTAACAAAAGTCTCTCGGAGTCCAT**G**GGAGCaaaagttatc-3'
EGS13 5'-AATTTAATACGACTCACTATTAAAAAAGTCTGCGCCAGCTACAT**G**GGAGCaaaagttatc-3'
EGS14 5'-AATTTAATACGACTCACTATTAAAGTCTCCCTATGTGGGCACAT**G**GGAGCaaaagttatc-3'
EGS15 5'-AATTTAATACGACTCACTATTAAGCAAAAGTGCCGTAGCGTCAT**G**GGAGCaaaagttatc-3'
EGS16 5'-AATTTAATACGACTCACTATTAAGCAAAAGTCCCCCAATTTCAT**G**GGAGCaaaagttatc-3'
EGS17 5'-AATTTAATACGACTCACTATTAAAAAAGTCTCCTTAGAGTTCAT**G**GGAGCaaaagttatc-3'
EGS18 5'-AATTTAATACGACTCACTATTAACAAAAGTCCCCCCAGGGACAT**G**GGAGCaaaagttatc-3'
EGS19 5'-AATTTAATACGACTCACTATTAAAAAGTCTCCCTCGTAGGGCAT**G**GGAGCaaaagttatc-3'
EGS20 5'-AATTTAATACGACTCACTATTAAGCAAAAGTACAACTGGCCCAT**G**GGAGCaaaagttatc-3'
EGS21 5'-AATTTAATACGACTCACTATTAAAAAAGTCTTTGATGGAAGCAT**G**GGAGCaaaagttatc-3'
EGS22 5'-AATTTAATACGACTCACTATTAAGCAAAAGTAAGTCTTTATCAT**G**GGAGCaaaagttatc-3'
EGS23 5'-AATTTAATACGACTCACTATTAAGCAAAAGTGTGTACCCGGCAT**G**GGAGCaaaagttatc-3'
EGS24 5'-AATTTAATACGACTCACTATTAAAAGTCTCCCCGTCTTTTTCAT**G**GGAGCaaaagttatc-3'
EGS25 5'-AATTTAATACGACTCACTATTAAGCAAAAGTGTTTAGTTTTCAT**G**GGAGCaaaagttatc-3'
EGS26 5'-AATTTAATACGACTCACTATTAAGCAAAAGTTGGGGGGGACCAT**G**GGAGCaaaagttatc-3'
EGS27 5'-AATTTAATACGACTCACTATTAAAAAAGTCTCGTGTCTGGACAT**G**GGAGCaaaagttatc-3'
EGS28 5'-AATTTAATACGACTCACTATTAACAAAAGTCCAAAGTGCTGCAT**G**GGAGCaaaagttatc-3'
EGS29 5'-AATTTAATACGACTCACTATTAAAGTCTCCCCATGGGGGCGCAT**G**GGAGCaaaagttatc-3'
EGS30 5'-AATTTAATACGACTCACTATTAAGCAAAAGTTAAAAGTCCTCAT**G**GGAGCaaaagttatc-3'
EGS31 5'-AATTTAATACGACTCACTATTAAGCAAAAGTGCTCTCCCCGCAT**G**GGAGCaaaagttatc-3'
EGS32 5'-AATTTAATACGACTCACTATTAACAAAAGTCAATGTTGGGCCAT**G**GGAGCaaaagttatc-3'
EGS33 5'-AATTTAATACGACTCACTATTAAAGTCTCCCAGGGCGTGTACAT**G**GGAGCaaaagttatc-3'
EGS34 5'-AATTTAATACGACTCACTATTAAAAGTCTCCTTTAAAGGGTCAT**G**GGAGCaaaagttatc-3'
EGS35 5'-AATTTAATACGACTCACTATTAAGCAAAAGTGCCAGGGCACCAT**G**GGAGCaaaagttatc-3'
