# Supplementary material for: Repair of Mutated NF1 mRNA with Trans-Splicing Group I Intron Ribozymes
Source: Cancers (Basel). 2025 Aug 23;17(17):2749. doi: 10.3390/cancers17172749 (PMC12427287; doi:10.3390/cancers17172749)
Supplement: Supplementary file 1 [file cancers-17-02749-s001.zip › Table S2. RzSequencesTargetingSpliceSites.pdf]

Table S2. Sequences of RNA constructs and PCR primers for *in vitro* reactions.

501 nucleotide long fragment of *mNf1* mRNA used for trans-splicing on splice sites 6577 (red), 6580 (blue), and 6582 (purple).

TATCTCTTCCATGTTGTCACTTTCTTAGTAGCCACAGGTCCCTTGTCCCTCCGAGCTTCCACACATGGGCTGCTCATCA
ATATCATTCACTCTCTGTGTACTTGTTCTCAGCTTCACTTTAGTGAAGAGACCAAGCAAGTTTTGAGGCTCAGTCTAAC
AGAGTTCTCGTTACCCAAATTTTACTTACTGTTTGGCATTAGCAAAGTCAAGTCGGCTGCTGTCATTGCCTTCCGTTCC
AGTTACCGGGACCGCTCCTTCTCCCCTGGC**T**CC**T**A**T**GAGAGGGAGACTTTTGCTTTGACGTCCCTGGAAACAGTCACAG
AAGCTTTGTTGGAGATCATGGAGGCATGTATGAGAGATATTCCAACATGCAAGTGGCTGGATCAGTGGACAGAACTAGC
TCAAAGATTTGCGTTTCAGTATAACCCATCGCTGCAGCCAAGAGCTCTTGTGGTGTTTGGCTGTATTAGCAAACGAGTG
TCTCATGGGCAGATAAAGCAGATTATC

524 nucleotide long fragment of *mNf1* mRNA used for trans-splicing on splice site 6355 (green). The substrate for splice site 6355 was different than for the other three splice sites to allow the splice site to be flanked by more than 200 nucleotides of sequence and therefore provide a closer model to the natural context in *mNf1* mRNA.

GAGGCTTAGGGTCTATCAAAGCTGAGGTGATGGCAGACACAGCTGTGGCTTTAGCTTCTGGAAATGTGAAATTGGTGTC
GAGTAAGGTTATTGGAAGGATGTGTAAAATAATTGACAAGACTTGCTTATCCCCAACTCCAACTTTAGAACAACATCTT
ATGTGGGACGACATTGCCATTTTAGCCCGCTACATGCTGATGCTGTCCTTCAACAACTCCCTCGATGTGGCGGCTCATC
TGCCCTATCTCTTCCATGTTGTCACTTTCTTAGTAGCCACAGGTCCCTTG**T**CCCTCCGAGCTTCCACACATGGGCTGCT
CATCAATATCATTCACTCTCTGTGTACTTGTTCTCAGCTTCACTTTAGTGAAGAGACCAAGCAAGTTTTGAGGCTCAGT
CTAACAGAGTTCTCGTTACCCAAATTTTACTTACTGTTTGGCATTAGCAAAGTCAAGTCGGCTGCTGTCATTGCCTTCC
GTTCCAGTTACCGGGACCGCTCCTTCTCCCCTGGCTCCTATGAGAGGGAG

Ribozyme DNA templates were obtained by PCR from a plasmid containing the *Tetrahymena* ribozyme body

aaaagttatcaggcatgcacctggtagctagtctttaaaccaatagattgcatcggtttaaaaggcaagaccgtcaaat
tgcgggaaaggggtcaacagccgttcagtaccaagtctcaggggaaactttgagatggccttgcaaagggtatggtaat
aagctgacggacatggtcctaaccacgcagccaagtcctaagtcaacagatcttctgttgatatggatgcagttcacag
actaaatgtcggtcggggaagatgtattcttctcataagatatagtcggacctctccttaatgggagctagcggatgaa
gtgatgcaacactggagccgctgggaactaatttgtatgcgaaagtatattgattagttttggagtactcg-3'

The templates for ribozymes testing the four different splice sites were generated using the following 5' PCR primers, containing either the promoters for T7 RNA polymerase (blue) in the version phi 6.5 promoter (ending in A) when transcription started with G, or in the version of the phi 2.5 promoter (ending in T) when transcription started with A. In cases where transcription would have started with a pyrimidine, two As were added to facilitate reasonable transcription efficiency. The first three nucleotides of the P1ex (green), the desired IGS (**G**XXXXX) and the first 20 nucleotides of the Tetrahymena ribozyme body (underlined) are indicated.

5'-AATTTAATACGACTCACTATAGGG**G**CAAGGaaaagttatcaggcatgcac-3'
5'-AATTTAATACGACTCACTATTAGG**G**GCCAGaaaagttatcaggcatgcac-3'
5'-AATTTAATACGACTCACTATTAACAT**G**GGAGCaaaagttatcaggcatgcac-3'
5'-AATTTAATACGACTCACTATTAACTC**G**TAGGAaaaagttatcaggcatgcac-3'

The reverse primer adding the ribozyme 3'-tail as shown in figure 1.

5'-GactgGTgAggGCgAAgGTtTCgCgtTCATcgagtactccaaaactaatc-3'
