# Supplementary figures and images for: Repair of Mutated NF1 mRNA with Trans-Splicing Group I Intron Ribozymes
Source: Cancers (Basel). 2025 Aug 23;17(17):2749. doi: 10.3390/cancers17172749 (PMC12427287; doi:10.3390/cancers17172749)

blots in Figure 3B

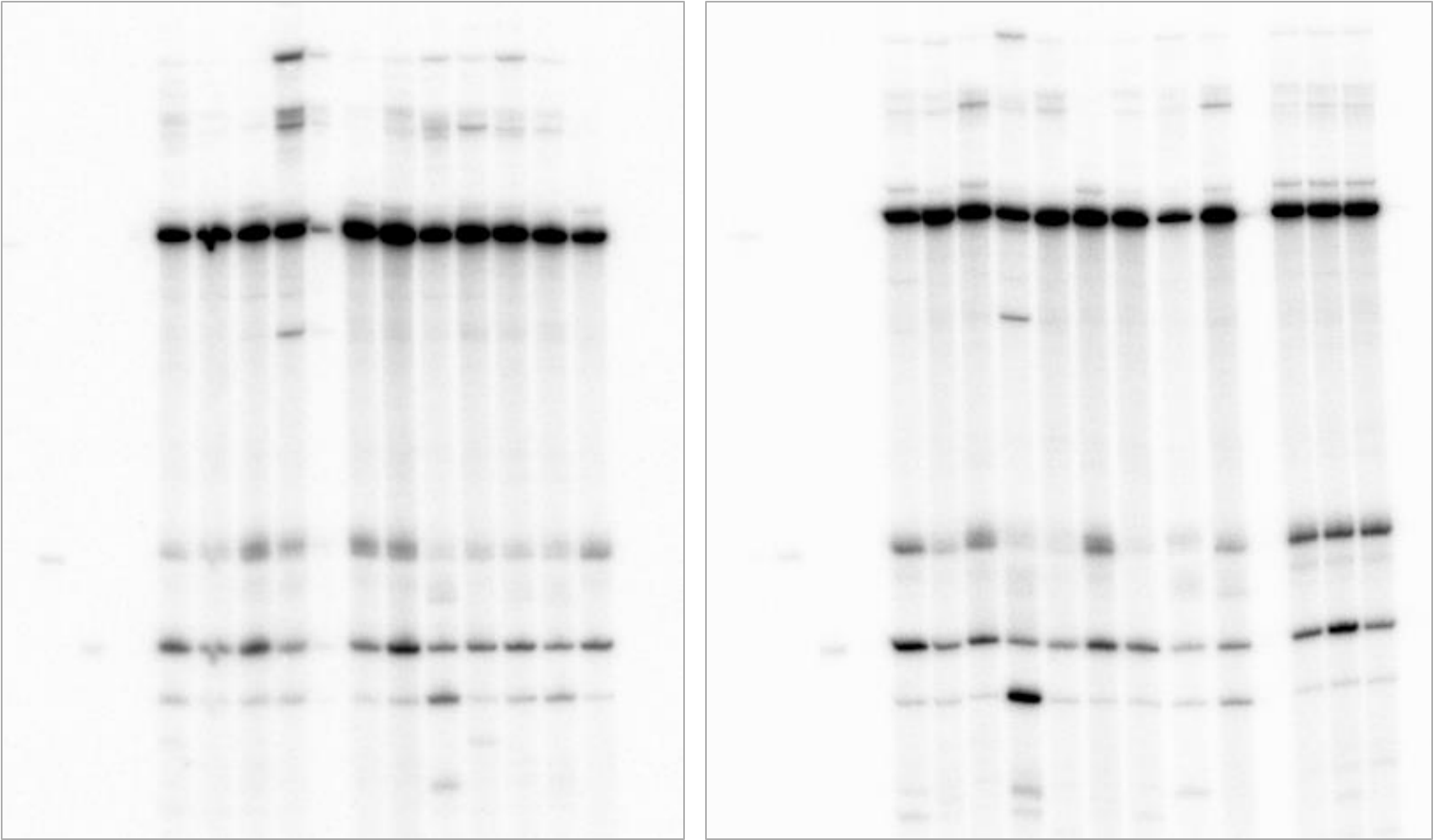

blots in Figure 5A

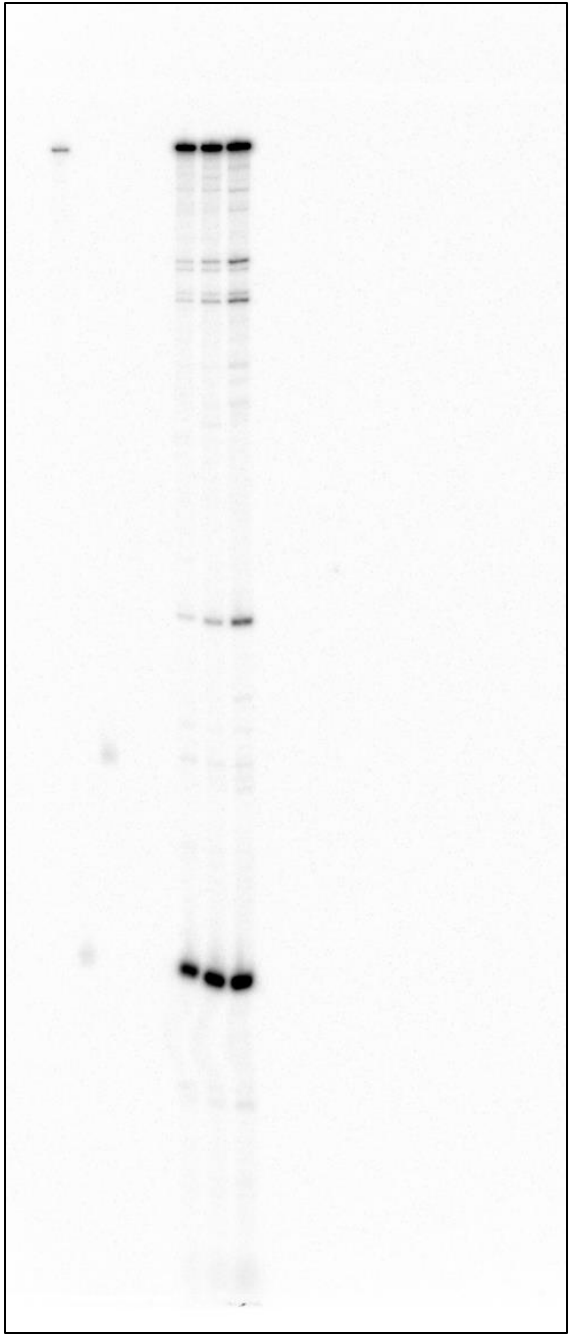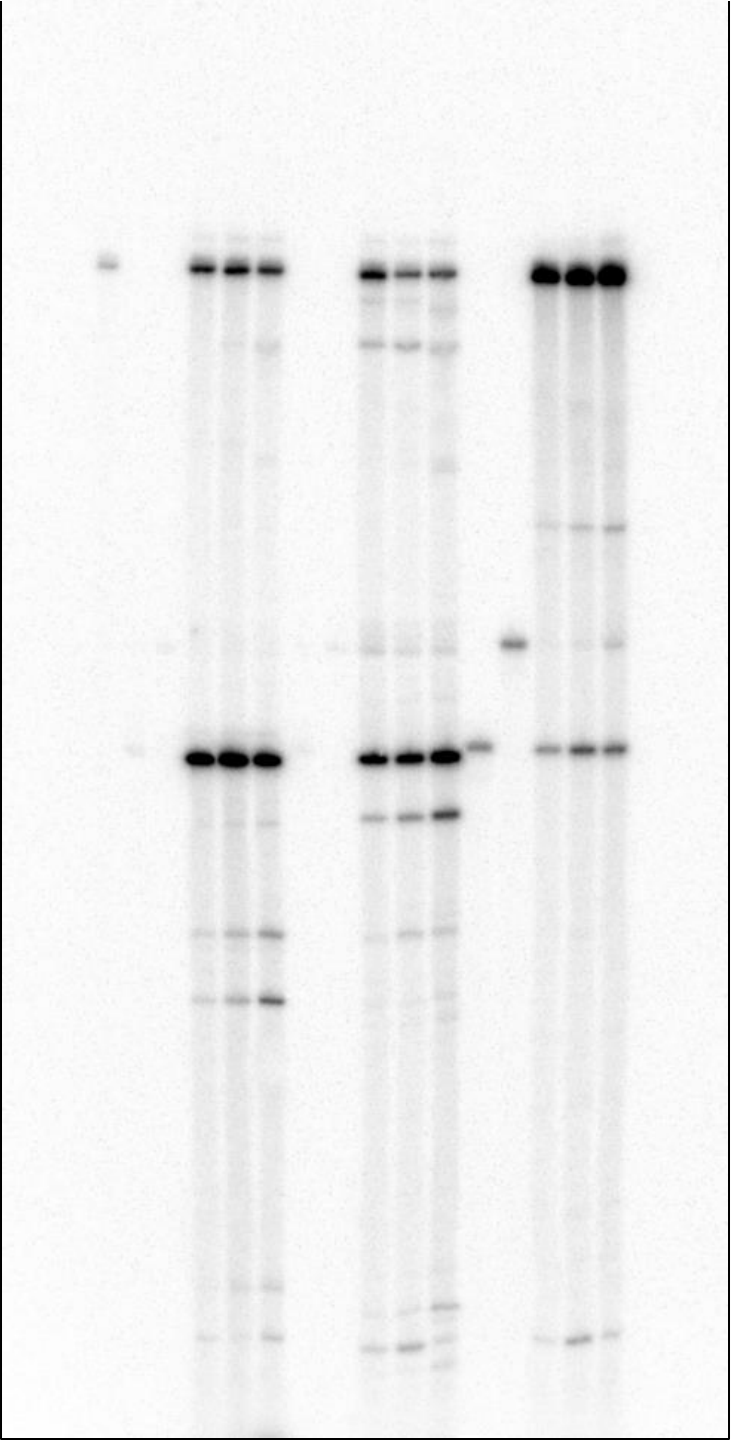

Supplement: Supplementary file 1 [file cancers-17-02749-s001.zip › File S1. Full pictures of the blots in Figures 3B and 5A.pdf]
